# Translatability of Scalp EEG Recordings of Duration-Deviant Mismatch Negativity Between Macaques and Humans: A Pilot Study

**DOI:** 10.3389/fpsyt.2020.00874

**Published:** 2020-08-26

**Authors:** Mariko Tada, Yuki Suda, Kenji Kirihara, Daisuke Koshiyama, Mao Fujioka, Kaori Usui, Tsuyoshi Araki, Kiyoto Kasai, Takanori Uka

**Affiliations:** ^1^Department of Neuropsychiatry, Graduate School of Medicine, University of Tokyo, Tokyo, Japan; ^2^International Research Center for Neurointelligence (IRCN), Bunkyo, Japan; ^3^Department of Integrative Physiology, Graduate School of Medical, University of Yamanashi, Yamanashi, Japan; ^4^Brain Science Institute, Tamagawa University, Machida, Japan

**Keywords:** mismatch negativity, schizophrenia, electroencephalogram (EEG), macaque, animal model

## Abstract

Mismatch negativity (MMN) is a negative deflection of the auditory event-related potential (ERP) elicited by an abrupt change in a sound presented repeatedly. In patients with schizophrenia, MMN is consistently reduced, which makes it a promising biomarker. A non-human primate (NHP) model of MMN based on scalp electroencephalogram (EEG) recordings can provide a useful translational tool, given the high structural homology of the prefrontal and auditory cortices between NHPs, such as macaques, and humans. However, in previous MMN studies, the NHP models used did not allow for comparison with humans because of differences in task settings. Moreover, duration-deviant MMN (dMMN), whose reduction is larger than that in the frequency-deviant MMN (fMMN) in patients with schizophrenia, has never been demonstrated in NHP models. In this study, we determined whether dMMN can be observed in macaque scalp EEG recordings. EEGs were recorded from frontal electrodes (Fz) in two Japanese macaques. Consistent with clinical settings, auditory stimuli consisted of two pure tones, a standard and a deviant tone, in an oddball paradigm. The deviant and standard tones differed in duration (50 and 100 ms for the standard and deviant tones, respectively). A robust dMMN with a latency of around 200 ms, comparable to that in humans, was observed in both monkeys. A comparison with fMMN showed that the dMMN latency was the longer of the two. By bridging the gap between basic and clinical research, our results will contribute to the development of innovative therapeutic strategies for schizophrenia.

## Introduction

Abnormal auditory information processing is a key feature of schizophrenia (1). Mismatch negativity (MMN) is an electrophysiological response, generally elicited in an auditory oddball paradigm (2). Given its reliability across repeated experiments, MMN is a promising biomarker (3, 4). For example, MMN amplitude reduction is one of the most robust of several neurophysiological and neurocognitive biomarkers in patients with schizophrenia (5). Moreover, recent studies have shown that MMN is also reduced in patients at clinical high-risk (CHR) for psychosis (6–15), and that an MMN reduction in CHR predicts conversion to psychosis (16, 17). The relationship of MMN to the functional abilities of patients, assessed using the Global Assessment of Functioning Scale (GAF) (3, 18–23), for example, is also of clinical value.

MMN has been explored not only in the clinical setting, but also in basic research. As a translatable brain marker (24, 25), MMN can aid the development of new therapies, and facilitate studies of the pathophysiology of schizophrenia. MMN is elicited passively in the auditory oddball paradigm and does not require a behavioral response, which enables identical physiological activities to be monitored in experimental animals and patients. MMN has been examined in animal studies, including rodent (reviewed in (25, 26), cat (27, 28), guinea pig (29), rabbit (30), and non-human primate (NHP) [reviewed in (25)] models. The major advantage of NHPs is the high homology with humans, especially in the structure of the prefrontal and auditory cortices, both of which are essential for MMN generation (31). Following the pioneering work of Javitt and colleagues (32), several groups have investigated MMN in NHPs.

Despite the unique findings regarding MMN generation obtained in animal studies, their relevance to clinical MMN studies is unclear because of differences in the auditory paradigms used (i.e., sequence of tone stimuli, deviant stimulus type, inter-stimulus interval and proportion of deviant stimuli in the oddball paradigm). For comparative studies of animal models and humans, the auditory task should be the same. In conventional clinical studies, there are two types of auditory stimuli: a frequent, standard stimulus and an infrequent, deviant stimulus. The deviant stimuli commonly used in schizophrenia assessments are changes in the frequency and duration of pure tones. While a few studies have used the two-tone oddball task in NHP models, and both intensity-deviant (32–35) and frequency-deviant MMN (fMMN) (36–38) studies have been reported for NHPs, this is not the case for duration-deviant MMN (dMMN). However, in a meta-analysis (39), the effect size in patients with schizophrenia was significantly larger for dMMN reduction (0.94; confidence interval [CI] = 0.85–1.04) than for fMMN reduction (0.72; CI = 0.57–0.87). Whether a dMMN reduction predicts the onset of psychosis in CHR has also been considered (16).

In this study, we determined whether dMMN could be obtained in scalp electroencephalogram (EEG) recordings from awake macaque monkeys. We used an auditory oddball task with a long-duration deviant, to allow comparison with the results of our previous clinical MMN studies (13, 23, 40–42) demonstrating the validity of MMN reduction in patients with schizophrenia. We also recorded the fMMN and compared its waveform characteristics to those of the dMMN.

## Methods

### Subjects

EEGs were recorded in two male Japanese monkeys (monkeys F and N; *Macaca fuscata*, 5.0–6.6 kg). The monkeys were trained to sit calmly in a chair before they underwent head-post implantation, using standard aseptic surgical procedures, to allow head fixation during the experiment. Recordings were obtained at Tamagawa University and the University of Yamanashi. All animal care and experimental procedures used in this study were approved by Tamagawa University and the University of Yamanashi Animal Care and Use Committees, and were performed in accordance with the National Institutes of Health guidelines.

In addition, an EEG was recorded in a healthy human (30-year-old male) using the same EEG acquisition device to allow direct comparison between monkeys and humans. The participant was confirmed to have no hearing impairment, psychiatric/neurological condition, or first-degree relative with schizophrenia. This recording study was approved by the Ethics Committee of the Faculty of Medicine, University of Tokyo [approval no. 629-(18)].

### Auditory Stimuli and EEG Data Acquisition Procedures

**Figure 1:**
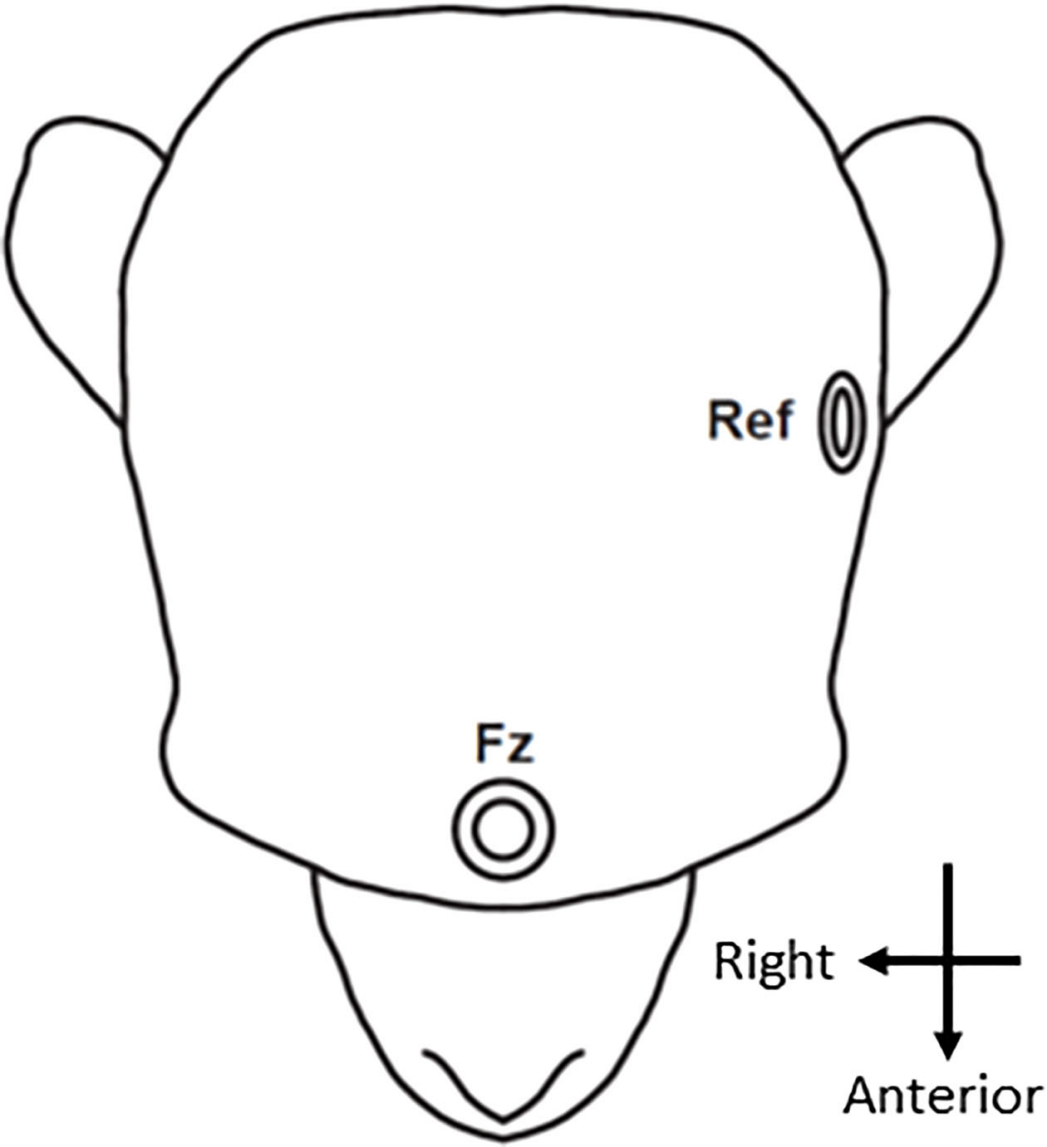
Electrode location. The EEG signal was recorded from an electrode positioned in the frontal area (Fz). The reference electrode (Ref) was placed in front of the ear.

Two types of auditory oddball paradigm, both identical to those used in our previous clinical studies (13, 23, 40–42) in monkeys and humans, were employed in this study. In the monkeys, the dMMN was based on the two-tone auditory oddball paradigm and consisted of 1,000 stimuli (standard tones: 1,000 Hz, 50 ms, 90%; deviant tones: 1,000 Hz, 100 ms, 10%); in the fMMN, the 1,000 stimuli consisted of a standard tone (1,000 Hz, 50 ms, 90%) identical to that in the dMMN condition, and a deviant tone (1,200 Hz, 50 ms, 10%). Monkey F underwent 8 duration-deviant and 8 frequency-deviant sessions, while monkey N underwent 13 sessions of each. Previous macaque studies showed that monkeys were capable of discriminating differences well below 100 Hz at the 1,000 Hz tone frequency (43, 44), and that the Weber fraction for tone duration was 0.42 at 50 ms (i.e., monkeys can discriminate between 50 and 71 ms) (45). Identical tone stimuli were presented to the human participant over 1,000 trials in one session. The “flip-flop” control paradigm (46, 47) was conducted in monkey F. Here, the standard and deviant tones were reversed compared to the original oddball paradigm so that the 1,000 stimuli were as follows (standard tones: 1,000 Hz, 100 ms, 90%; deviant tones: 1,000 Hz, 50 ms, 10%). Monkey F underwent 8 flip-flop sessions. The auditory stimuli were provided at a sound pressure level of 80 dB, with a 1-ms rise/fall time. The onset of stimulus asynchrony was at 500 ms. Auditory stimuli were presented binaurally using earphones identically inserted in the monkeys and human. The two oddball conditions were counterbalanced. During the experiment, the monkeys sat in a primate chair positioned in a quiet, electrically shielded room. Monkey N was trained on a fixation task to minimize eye movement artifacts. A Polymate II-AP216 EEG, active electrode system (Miyuki Giken Co., Ltd., Tokyo, Japan) was used to record the EEGs. The electrode (Au) was manually placed on the frontal scalp just anterior to the head post, and was referenced to within 1 cm of the tragus of the left ear (**Figure 1**), because the mastoid area was not accessible due to the head-post surgery. The ground electrode was located just above the external occipital protuberance. In the human, a frontal electrode (Fz) was also used for the recording. The reference electrode was located at the left mastoid and the ground electrode was located at the right mastoid in keeping with a previous multi-site study (48). Impedance was below 20 kΩ, the sampling rate was 1,000 Hz, and the analog filter bandpass was set at 0.5–100 Hz.

### Data Analysis

Off-line analyses were performed using EEGLAB (49), as described in our previous clinical studies (13, 23, 40–42). Continuous EEG data from the Fz electrode were digitally filtered at 3 to 20 Hz and segmented from −100 to 500 ms relative to stimulus onset. The mean of the pre-stimulus baseline was subtracted for baseline correction. Epochs exceeding ±50 μV were rejected. As a result, there were 3,924 out of 7,200 standard and 438 out of 800 deviant trials in monkey F, 5,977 out of 11,700 standard and 680 out of 1300 deviant trials in monkey N, and 899 out of 900 standard and 100 out of 100 deviant trials in human. The ERPs to the standard and deviant stimuli were calculated as mean values across trials, and the differential waveform was calculated by subtracting the standard response from the deviant response at each time point. Peak time latencies were used to evaluate waveform characteristics.

All statistical analyses were conducted using custom scripts written in MATLAB R2014a (MathWorks, Natick, MA, USA). MMN was evaluated using two-sample *t* tests (two-tailed) by comparing the trial-wise responses to the standard and deviant stimuli at each time point (every 1 ms from 100 ms before to 499 ms after tone onset). The false discovery rate (FDR, Benjamini-Hochberg method) was used to adjust for comparisons across multiple time points.

## Results

### Duration and Frequency of MMN in Monkeys

**Figure 2:**
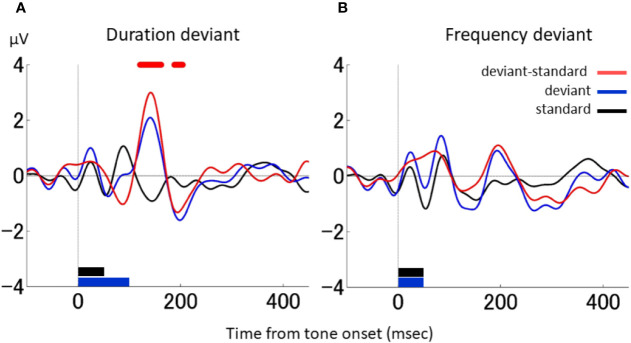
dMMN and fMMN in macaque monkeys. **(A)**, Grand average waveforms in the duration-deviant experiment conducted on two macaque monkeys. The electrical potential is plotted as a function of the time from tone onset. The black line shows the responses to the standard stimulus, the blue line the responses to the deviant stimulus, and the red line the differential waveform. The stimulus duration (indicated at the bottom of the graph) was 50 ms for the standard stimulus (black) and 100 ms for the deviant stimulus (blue). The stimulus frequency was 1,000 Hz for both the standard and deviant stimuli. Red dots on the top of the graph denote the times at which the responses to the standard and deviant stimuli were statistically different (two-sample t test, p < 0.05, FDR corrected). **(B)**, Grand average waveforms in the frequency-deviant experiment conducted on two macaque monkeys. The stimulus frequency (shown at the bottom of the graph) was 50 ms for both the standard (black) and deviant (blue) stimuli. The stimulus frequency was 1,000 Hz for the standard stimulus and 1,200 Hz for the deviant stimulus. There were no statistical differences between the responses to the standard and deviant stimuli. The figure conventions are the same as in A.

**Figure 2A** shows the grand average waveforms obtained from the two macaque monkeys during the duration-deviant experiment. The black line indicates the responses to the standard stimulus, the blue line the responses to the deviant stimulus, and the red line the differential waveform. The triphasic pattern in the differential waveform showed an initial negative deflection around 100 ms (peak latency 88 ms; −1.03 μV), followed by a positive deflection around 150 ms (peak latency 143 ms; 3.00 μV) and a second negative deflection around 200 ms (peak latency 195 ms; −1.32 μV). The ERPs to the standard and deviant stimuli were significantly different between the positive deflection (from 121 to 163 ms) and second negative deflection (from 188 to 205 ms) (two-sample *t*-test, *p* < 0.05, FDR corrected; *t*[11,017] > 2.82). The latency of the first negative deflection was similar to that reported for various types of MMN in NHPs (32–38). The latency of the second negative deflection was compatible with the dMMN reported in humans (13, 23, 40–42).

For comparison, **Figure 2B** shows the grand average waveforms obtained during the frequency-deviant experiment conducted in the macaques. The differential waveform showed a single negative deflection around 130 ms (peak latency 128 ms; −0.53μV), but it was not significant. The latency of this negative deflection was comparable to the fMMN reported in humans (13, 23, 40, 41). Therefore, human-compatible MMNs were observed in the monkey in both the duration- and frequency-deviant experiment, with each MMN producing prototypical waveforms.

### Evaluation of the Waveform Characteristics of dMMN and fMMN

**Figure 3:**
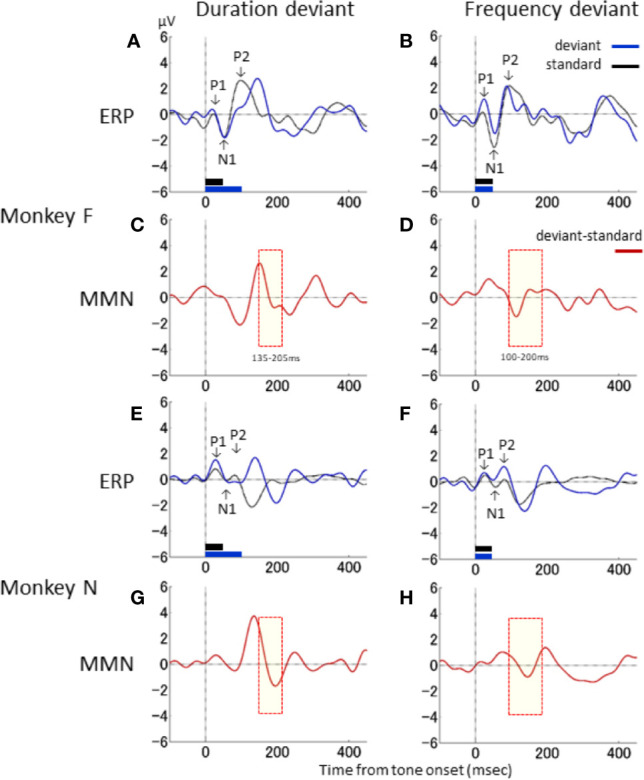
Waveform characteristics of ERP. Responses to the standard (black) and deviant (blue) stimuli, and the differential waveform (red), are shown for the duration-deviant **(A, C, E, G)** and frequency-deviant **(B, D, F, H)** experiments conducted on monkey F **(A–D)** and monkey N **(E–H)**. Conventions are the same as in . The yellow shaded area denotes the time window of the dMMN **(C, G)** and fMMN **(D, H)** in Nagai et al. (13).

Next, the waveform characteristics of the ERPs associated with the standard and deviant stimuli in the duration- and frequency-deviant experiments were evaluated. **Figure 3** shows the ERP and differential waveforms for the duration- and frequency-deviant experiments conducted on the two monkeys. The ERP to the standard stimulus was characterized by the typical P1–N1–P2 complex (**Figures 3A, B, E, F**). The P1 and N1 waveforms were mostly identical between the standard and the deviant stimuli in both the duration- and frequency-deviant experiment. However, the P2 response to the deviant stimulus was delayed compared to the P2 response to the standard stimulus in the duration-deviant, but not the frequency-deviant, experiment. Consequently, the differential waveform in the duration-deviant experiment had an initial negative deflection followed by a positive deflection (**Figures 3C, G**), as described in the previous section.

The latencies of the deflections in the differential waveforms were compared to the MMN latencies reported in our previous clinical studies (13, 23, 40–42). In **Figure 3**, the time windows of the human dMMN and fMMN are shaded in yellow (dMMN, 135–205 ms: **Figures 3C, G**; fMMN, 100–200 ms: **Figures 3D, H**). The latency of the second negative deflection in the duration-deviant experiment was compatible with the human dMMN. Similarly, the negative deflection in the frequency-deviant experiment was compatible with the human fMMN. The differential waveforms in the duration- and frequency-deviant experiments were roughly similar between the two monkeys (duration: **Figures 3C, G**; frequency: **Figures 3D, H**).

### Tone Duration Controlled MMN in the “Flip-Flop” Paradigm

The above-described duration-deviant experiment was performed using different tone durations, and the difference in stimuli might have affected the differential waveforms (specifically, the delayed P2). We therefore performed a control study wherein the standard and deviant tones were reversed in one monkey (monkey F). In this “flip-flop” paradigm (46, 47), the standard tone was 100 ms, and the deviant tone was 50 ms.

**Figure 4:**
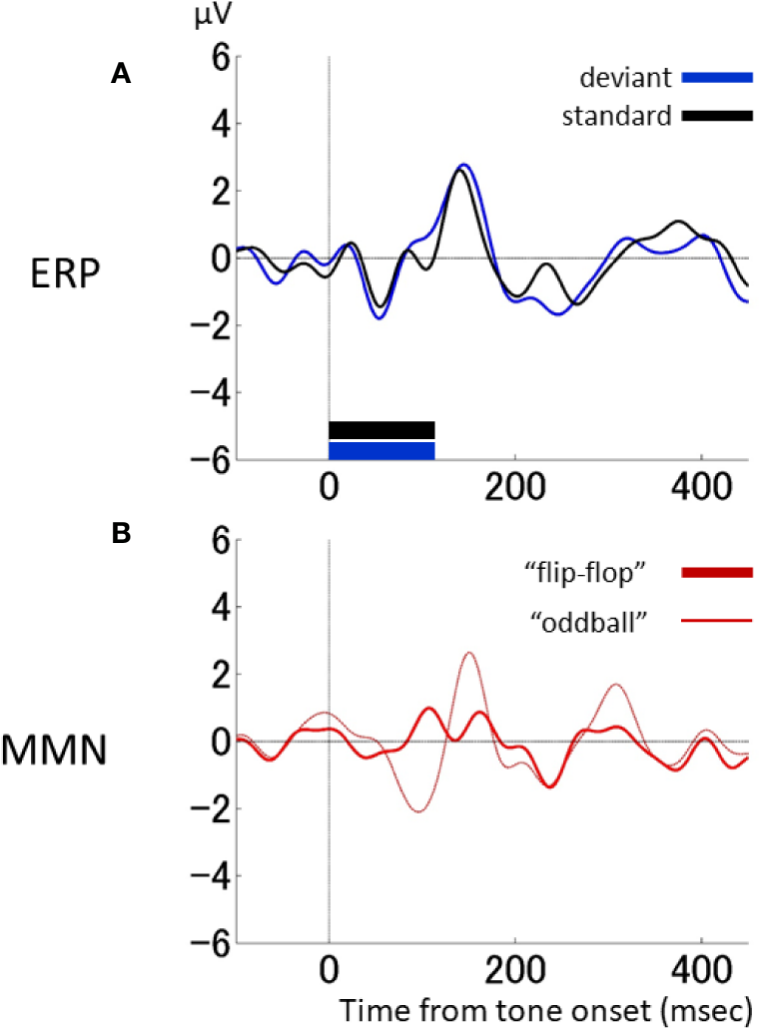
Responses to 100-ms-duration stimuli in different contexts. **(A)**, Response by one macaque monkey (monkey F) to 100-ms stimuli in a standard context (standard condition in the “flip-flop” paradigm: black) and a deviant context (deviant condition in the original oddball paradigm: blue). **(B)**, Comparison of differential waveforms using ERPs to the standard stimulus in the original oddball paradigm (thin line: same as ) and ERPs to the standard stimulus in the flip-flop paradigm (thick line).

**Figure 4** compares ERP responses to identical 100-ms tones in different contexts. Here, the ERP responses to the standard tone in the flip-flop paradigm (black in **Figure 4A**) and the deviant tone in the original paradigm (black in **Figure 4A**) both had a delayed P2. Thus, the corresponding differential waveform (thick line in **Figure 4A**) had a much smaller first negative deflection and positive deflection compared to the original differential waveform (thin line in **Figure 4B**). In contrast, the second negative deflection did not differ between the two waveforms. We therefore conclude that the delayed P2 and corresponding first negative and positive deflections observed in the original differential waveform were due to the difference in tone duration. Furthermore, the second negative deflection in the original duration-deviant experiment may be considered an MMN-like response.

### Comparison of Monkey and Human dMMN

**Figure 5:**
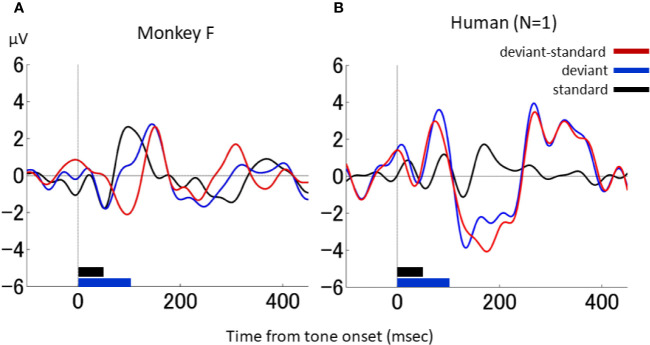
dMMN in macaque monkeys and in a human. **(A)**, Waveforms in the duration-deviant experiment from one macaque monkey (monkey F). **(B)**, Waveforms in the duration-deviant experiment from one human. Conventions are the same as in.

A direct comparison of the results of the NHP model with those obtained in the human is provided in **Figure 5**, which shows the differential waveforms during the duration-deviant experiment in one monkey (monkey F) and the human participant, obtained using the same EEG acquisition device (Polymate II-AP216). A similar triphasic pattern, consisting of a negative deflection around 100 ms followed by a positive deflection around 150 ms and a second negative deflection around 200 ms, was obtained in both the monkey and the human.

## Discussion

Although previous studies have reported MMN-like responses in NHPs, this is the first NHP study to report an MMN-like response during a duration-deviant experiment. Specifically, we aimed to determine whether a dMMN would occur in awake macaque monkeys subjected to an auditory oddball task compatible with that used in our previous clinical MMN studies (13, 23, 40–42). We observed a triphasic MMN-like response in the duration-deviant oddball paradigm, and confirmed that the second negative deflection was not due to differences in tone duration using the flip-flop paradigm. This suggests that the second negative deflection was an MMN-like response, although we cannot preclude the possibility that the first negative deflection was also an MMN-like response. On the other hand, a triphasic MMN has previously been illustrated as an MMN-P3a-RON chain (50). Although the latencies seem to suggest otherwise, this is a possibility if we assume that neural latencies are shortened considerably in the monkey brain. Further studies are necessary to investigate this possibility.

In addition, an fMMN in the monkeys was recorded, and its waveform characteristics were compared to those of the dMMN. In both the duration- and frequency-deviant experiment, an MMN-like negative deflection was elicited with a latency compatible to those of the dMMN and fMMN in humans (13, 23, 40–42). In previous NHP studies, the negative deflection around 100 ms was interpreted as an MMN-like response. Gil-da-Costa and colleagues (33) reported an MMN-like response at 100 ms after tone onset using an intensity-deviant paradigm. The latency of the fMMN (around 130 ms) in our study was comparable. The latency of the second negative deflection in the dMMN was longer, but that may be explained by the difference in auditory stimulus; both frequency and intensity differences can be detected at tone onset, but duration differences can only be detected after 50 ms at the earliest.

Because deviant-type effects have been examined in previous clinical studies, we also investigated the difference between dMMN and fMMN in monkeys. Not only the MMN latencies, but also their forms, were similar between the monkeys and the human: the dMMN had a sharp deflection, whereas the fMMN had a shallow, trapezoidal shape [**Figure 4** in (13)]. We further observed a delay in the P2 response to the deviant stimulus compared to the P2 response to the standard stimulus, but only in the duration-deviant, and not the frequency-deviant, experiment. The P2 latencies were around 100 ms, consistent with a previous study conducted on awake monkeys (7). The P2 latency was compatible with tone termination, such that tone offset detection (51) was presumably reflected in the P2 responses of the monkey. This was also consistent with the results from the flip-flop paradigm, in which the P2 was delayed for the 100ms standard stimulus. Indeed, a delay in the offset response has previously been observed more clearly in macaque than human (52). Nonetheless, the delayed P2 had a non-negligible effect on the waveform of the MMN-like response in the duration-deviant paradigm in the macaque.

Recognition of the different clinical characteristics of these two MMNs is critical if MMN is to be used as a biomarker of the early stages of schizophrenia. Our previous studies showed a significant reduction in the dMMN of patients with first-episode schizophrenia, and in CHR individuals, compared to healthy controls, whereas the fMMN reductions were not significant (13). These findings suggest that a reduction in dMMN reflects the pathophysiology of early stage illness or an altered developmental process; fMMN reduction is known to be associated with progressive brain changes and illness chronicity (24). In translational research, both dMMN and fMMN should be investigated to elucidate different mechanisms underlying the pathophysiology of schizophrenia. In the present study, identical EEG recording systems (including in terms of the auditory stimuli, earphones and EEG acquisition device) were used in the two monkeys and the human, allowing for direct comparison of their results. The probability of the deviant stimuli appearing in the sequence of tone stimuli is known to affect MMN amplitude; here we used a 10% probability, as in previous patient studies [reviewed in (24)]. Although careful comparisons are needed to determine whether MMN-like responses occur in experimental animals, our study demonstrated MMN-like responses, in terms of both duration and frequency, occurring within a similar time window in macaques and the human participant. Gil-da-Costa and colleagues (33) also used homologous scalp EEG acquisition systems, including noninvasive EEG caps, in monkeys and humans and obtained MMNs that were similar in terms of their latency and topography. Translational studies are needed before MMN can serve as a brain marker in preclinical and clinical studies. The present study could contribute to future translational investigations aiming to understand the neural mechanism of reduced dMMN in the early stage of schizophrenia, using dMMN as a marker for pharmacological or neurophysiological intervention in NHP models.

We were only able to record from Fz because the head-post was located at the top of the skull (i.e., the central electrode [Cz]). While the most robust MMN is obtained at the frontocentral electrode site (FCz), the amplitude of MMN is known to be reversed at the mastoids in human scalp EEG recordings. Therefore, in future NHP studies, EEGs should be recorded from a wider area. Indeed, a previous NHP study showed that the ERP amplitudes and latencies of the MMN-like response were topographically organized in temporal, frontal, and occipital electrodes under the frequency condition (53). Another previous study showed comparable topography of MMN between NHP and human (33). Moreover, because we could not record electrooculograms, we could determine whether slow potentials with low amplitude affected the results.

In conclusion, this study demonstrated homologous MMNs between macaque monkeys and humans in an auditory task identical to that applied in the clinical setting. By bridging the gap between basic and clinical research, our results will contribute to the development of innovative therapeutic strategies for patients with schizophrenia.

## Data Availability Statement

The raw data supporting the conclusions of this article will be made available by the authors, without undue reservation.

## Ethics Statement

The studies involving human participants were reviewed and approved by the Ethical Committee of the Faculty of Medicine, University of Tokyo. The patients/participants provided their written informed consent to participate in this study. The animal study was reviewed and approved by Tamagawa University and the University of Yamanashi Animal Care and Use Committees.

## Author Contributions

MT, YS, KKi, DK, and TU collected the data. MT, YS, KKi, and TU analyzed the data. MT, YS, KKi, DK, MF, KU, TA, KKa, and TU interpreted the results. MT, YS, KKi, TA, KKa, and TU designed the study. KKa and TU supervised all aspects of data collection, analysis, and interpretation. MT and TU wrote the manuscript. All authors contributed to the article and approved the submitted version.

## Funding

This work was supported in part by the Japan Society for the Promotion of Science (JSPS) KAKENHI (19K17105; MT; 18K07588; KKi), the Brain Mapping by Integrated Neurotechnologies for Disease Studies (Brain/MINDS) of the Japan Agency for Medical Research and Development (AMED) (JP20dm0207069; JP19dm0207069; KKa and TU), the International Research Center for Neurointelligence (WPI-IRCN) of the University of Tokyo Institutes for Advanced Study (UTIAS) (MT, KKa), the University of Tokyo Center for Integrative Science of Human Behavior (CiSHuB) (KKa), the SENSHIN Medical Research Foundation (MT), and the Takeda Science Foundation (MT).

## Conflict of Interest

The authors declare that the research was conducted in the absence of any commercial or financial relationships that could be construed as a potential conflict of interest.
